# Propensity score-matching analysis of postoperative radiotherapy for stage IIIA-N2 non-small cell lung cancer using the Surveillance, Epidemiology, and End Results database

**DOI:** 10.1186/s13014-017-0836-6

**Published:** 2017-06-13

**Authors:** Shenhai Wei, Mian Xie, Jintao Tian, Xiaoping Song, Bingqun Wu, Limin Liu

**Affiliations:** 1 Department of Thoracic Surgery, First Hospital of Tsinghua University, Beijing, China; 2 China State Key Laboratory of Respiratory Disease, The First Affiliated Hospital of Guangzhou Medical University, Guangzhou, China; 3 Department of Physiology, Capital Medical University, No.10, Xitoutiao, Youanmen, Beijing, 100069 People’s Republic of China

**Keywords:** Non-small cell lung cancer, Postoperative radiotherapy, Survival

## Abstract

**Background:**

To investigate the effects of postoperative radiotherapy (PORT) on the survival of patients with resected stage IIIA-N2 non-small cell lung cancer (NSCLC).

**Methods:**

A total of 3,334 patients with resected stage IIIA-N2 NSCLC in 2004 to 2013 were identified in the Surveillance, Epidemiology, and End Results database and stratified according to use of PORT. Propensity score-matching (PSM) methods were used to balance the baseline characteristics of patients who did (*n* = 744) or did not (*n* = 744) undergo PORT. Overall survival (OS) and lung cancer-specific survival (LCSS) were compared between these two patient groups.

**Results:**

After PSM, PORT increased OS (hazard ratio, 0.793; *p* = 0.001) and LCSS (hazard ratio, 0.837; *p* = 0.022) compared with no PORT. The OS benefit for PORT was mainly seen in patients aged <60 years (5-year OS, 35.4% versus 28.9% for PORT versus no PORT, respectively; *p* = 0.026) and in those who underwent lobectomy (5-year OS, 43.5% versus 34.5% for PORT versus no PORT, respectively; *p* = 0.001). The LCSS benefit for PORT was significant in patients undergoing lobectomy (5-year LCSS, 48.3% versus 42.3% for PORT versus no PORT, respectively; *p* = 0.036).

**Conclusions:**

The survival benefits of PORT were primarily observed in patients with resected stage IIIA-N2 NSCLC who were <60 years of age or had undergone lobectomy.

## Background

Lung cancer is the leading cause of cancer-related mortality among both men and women worldwide [1]. Most lung cancers are non-small cell lung cancers (NSCLCs) [1]. Although surgical resection remains the mainstay of therapy for NSCLC without metastasis, local relapse and distant metastasis can occur after surgery, especially at advanced disease stages. In patients with node-positive disease, for example, the risk of locoregional recurrence is as high as 20%–40% [2].

Postoperative radiotherapy (PORT) sterilizes regions at risk of microscopic disease and thus is an appealing means of preventing locoregional recurrence and improving outcomes in NSCLC patients. Studies on patients with stage I, stage II, or stage IIIA NSCLC have been performed to test this hypothesis [3–6]. These studies consistently showed detrimental effects of PORT on the survival of early-stage (stages I and II) patients [4, 6–9]. In contrast, the results for stage III patients with N2 NSCLC were conflicting. PORT had survival advantages in a randomized trial of adjuvant chemotherapy, in which the use of PORT was not randomized or mandatory [4], and in two population-based cohort studies, one using the National Cancer Data Base (NCDB) [5] and the other using the Surveillance, Epidemiology, and End Results (SEER) database [6]. On the other hand, in the randomized controlled trial conducted by Shen et al.[10], PORT decreased the incidence of local recurrence and distant metastasis, but failed to improve overall survival (OS) when administered after complete resection of N2 NSCLCs. PORT also failed to improve OS, as well as failure-free survival, in the earlier study by Perry et al. [11] on resected N2 NSCLC. The results of several meta-analyses are contradictory and hence do not justify the routine use of PORT in patients with completely resected N2 NSCLC [7, 9, 12, 13].

In this study, we explored the effects of PORT in patients with resected stage IIIA-N2 NSCLC using SEER data from 2004 to 2013 and propensity score-matching (PSM) methods.

## Methods

The SEER program collects data from 18 population-based registered cancer institutes that cover approximately 30% of the US population [14]. We used SEER*Stat version 8.3.2 software to extract data from the SEER database. This study was approved by the review board of our institute.

The selection criteria included adult patients (age ≥ 20 years) who underwent resection for pathologically confirmed NSCLC without distant metastasis between 2004 and 2013. To fulfill these inclusion criteria, we selected patients with adenocarcinoma (SEER codes 8140, 8250, 8252–8255, 8260, 8310, 8323, 8480, 8481, 8490, 8570, 8574), squamous cell carcinoma (SEER codes 8052, 8070–8074, 8083, 8084), large cell carcinoma (SEER codes 8012, 8013), and adenosquamous carcinoma (SEER code 8560). Only patients coded as stage T1–3 and N2 were included in this study; those without positive regional lymph nodes (LNs) were excluded. Patients with a previous malignant disease were also excluded.

**Fig. 1:**
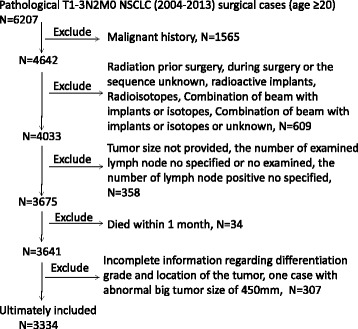
Patient selection for this study

Surgical types were categorized as sublobectomy, lobectomy, or pneumonectomy. Sublobectomy consisted of wedge resection and segmentectomy. Only patients who either underwent beam radiation after surgery or no radiation were included in this study. In an effort to account for surgical mortality, those who died within 1 month after surgery were excluded, as were those without complete information regarding tumor size, tumor location, regional LN examination results, histology, and differentiation grade. One case with an abnormally large tumor size (450 mm) was also excluded. Fig. 1 shows the detailed case selection process. Ultimately, our study consisted of 3,334 patients.

Data extracted for this study included age, sex, race, marital status, insurance coverage, laterality, tumor location, tumor size, T stage (based on the criteria of the 6th edition of the American Joint Committee on Cancer), histology, pathologic differentiation grade, surgical procedure, the number of examined LNs, the number of positive LNs, and the use of PORT. The ratio of positive to examined LNs was calculated for analysis as a continuous variable. Race, marital status, and insurance coverage were combined into dichotomized variables separately.

The endpoints were OS and lung cancer-specific survival (LCSS). OS was the time from diagnosis to death from any cause. LCSS was the time from diagnosis to death from lung cancer, and any deaths due to causes other than lung cancer were censored.

### Statistics

We used Pearson’s chi-square test to assess the association between the use of PORT and the categorical variables, and the Mann–Whitney *U* test to assess the association between the use of PORT and the continuous variables. Survival curves were generated by using the Kaplan-Meier method, and differences in survival among subgroups were examined by using the log-rank test. Multivariate Cox proportional hazards analysis was used to examine the association between survival and potential prognostic factors. In the pretest, we found that patients <60 years of age had a shorter survival time than those 60–79 years of age. Therefore, ages were grouped into three categories (<60 years, 60–79 years, and ≥80 years) in the multivariate analysis.

To balance the differences in the basic clinical characteristics between patients who underwent PORT and those who did not, we used PSM methods. Propensity scores were calculated via a logistic regression analysis including age, race, marital status, insurance coverage, laterality, tumor location, tumor size, T stage, histology, pathologic differentiation grade, surgical procedure, the number of LNs examined, the number of positive LNs, and the ratio of positive to examined LNs. Patients who received PORT and those who did not were matched 1:1 based on their propensity scores using nearest-neighbor matching, for which the matching tolerance was 0.01%. OS and LCSS were compared in patients who received PORT and those who did not by using the Kaplan-Meier method and Cox regression multivariate survival analysis was also performed to examine potential prognostic factors.

A probability value <0.05 was considered to be significant. All analyses were conducted by using SPSS version 22.0 software (SPSS Inc. Chicago, IL).

## Results

The patient cohort (*n* = 3,334) in this study consisted of 1655 men (49.6%) and 1679 women (50.4%) with a median age of 66.0 years (range, 22–93 years). Among these patients, 1,244 (37.3%) received PORT. The last follow-up occurred in December 2013, and the median follow-up duration was 24 months (range, 0–119 months). A total of 1,895 patients (56.8%) died during the follow-up period, and the median OS and LCSS times were 36 months and 43 months, respectively.

Married patients and patients with more positive LNs, a higher ratio of positive to examined LNs, poorer differentiation, or less resected lung tissue were more likely to receive PORT (Table 1). After adjusting for propensity scores, the patient and tumor characteristics were well balanced between the group that received PORT (*n* =744 patients) and the group that did not (*n* = 744) (Table 1).

**Table 1:** Demographics and clinical characteristics for patients treated with and without PORT before and after PSM

Demographic or clinical characteristic	Before PSM			After PSM		
	No PORT (*N *= 2090)	PORT (*N*=1244)	*P*	No PORT (*N *= 744)	PORT (*N *= 744)	*P*
Age, years (range)	65.5 (22–89)	65.7 (28–93)	0.581	66.4 (62–89)	65.7 (28–90)	0.692
Gender						
Male	1026 (62.0%)	629 (38.0%)	0.411	355 (48.2%)	381 (51.8%)	0.178
Female	1064 (63.4%)	615 (36.6%)		389 (51.7%)	363 (48.3%)	
Race						
White	1689 (62.8%)	999 (37.2%)	0.720	604 (49.9%)	607 (50.1%)	0.842
Nonwhite	401 (62.1%)	245 (37.9%)		140 (50.5%)	137 (49.5%)	
Marital status						
Married	1185 (59.4%)	810 (50.6%)	0.000	465 (50.6%)	454 (49.4%)	0.557
others	905 (67.6%)	434 (32.4%)		279 (49.0%)	290 (51.0%)	
Insurance						
Insured	1261 (61.7%)	783 (38.3%)	0.135	462 (49.5%)	471 (50.5%)	0.629
others	829 (64.3%)	461 (35.7%)		282 (50.8%)	273 (49.2%)	
Laterality						
Left	976 (63.9%)	552 (36.1%)	0.192	317 (47.7%)	347 (52.3%)	0.118
Right	1114 (61.7%)	692 (38.3%)		427 (51.8%)	397 (48.2%)	
Location						
Upper lobe	1229 (61.5%)	768 (38.5%)	0.084	436 (48.9%)	455 (51.1%)	0.587
Middle lobe	89 (58.9%)	62 (41.1%)		36 (52.9%)	32 (47.1%)	
Lower lobe	772 (65.1%)	414 (34.9%)		272 (51.4%)	257 (48.6%)	
Tumor size, cm (range)	3.8 (0.1–19.0)	3.7 (0.5–15)	0.340	3.7 (0.1–15)	3.8 (0.5–15)	0.670
LN positive (range)	3.4 (1–41)	3.8 (1–30)	0.000	3.4 (1–33)	3.5 (1–24)	0.062
LN examined (range)	12.2 (1–90)	11.6 (1–64)	0.006	11.9 (1–68)	12.0 (1–61)	0.473
% of LN positive (range)	35.4 (1.4–100)	41.2 (1.7–100)	0.000	35.5 (1.4–100)	35.8 (1.7–100)	0.380
Histology						
Adenocarcinoma	1423 (61.5%)	891 (38.5%)	0.097	478 (48.1%)	515 (51.9%)	0.110
Squamous cell carcinoma	505 (65.2%)	270 (34.8%)		204 (54.4%)	171 (45.6%)	
Adenosquamous and large cell carcinoma	162 (66.1%)	83 (33.9%)		62 (51.7%)	58 (48.3%)	
Differentiation						
Well differentiated	131 (72.4%)	50 (27.6%)	0.012	20 (40.8%)	29 (59.2%)	0.548
Moderately differentiated	930 (61.5%)	581 (38.5%)		354 (51.1%)	339 (48.9%)	
Poorly differentiated	969 (62.2%)	589 (37.8%)		354 (49.7%)	358 (50.3%)	
Undifferentiated	60 (71.4%)	24 (28.6%)		16 (47.1%)	18 (52.9%)	
Surgical procedure						
Sublobectomy	145 (51.4%)	137 (48.6%)	0.000	50 (51.0%)	48 (49.0%)	0.972
Lobectomy	1741 (62.9%)	1026 (37.1%)		643 (49.9%)	646 (50.1%)	
Pneumonectomy	204 (71.6%)	81 (28.4%)		51 (50.5%)	50 (49.5%)	
T stage (sixth edition)						
T1	629 (61.3%)	397 (38.7%)	0.359	235 (50.6%)	229 (49.4%)	0.908
T2	1341 (63.6%)	768 (36.4%)		468 (49.8%)	471 (50.2%)	
T3	120 (60.3%)	79 (39.7%)		41 (48.2%)	44 (51.8%)	

*LN* lymph node, *PSM* propensity score-matching, *PORT* postoperative radiotherapy

**Fig. 2:**
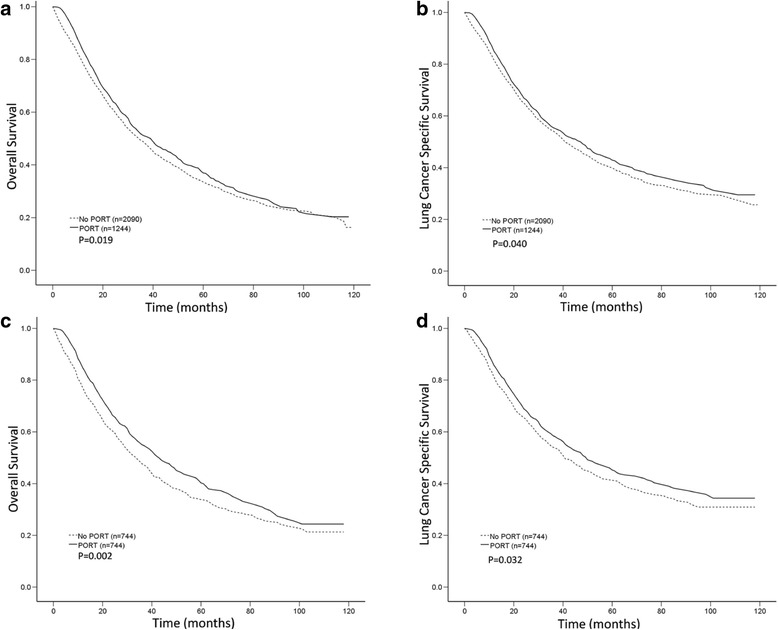
Overall survival (OS) and lung cancer-specific survival (LCSS) according to the use of postoperative radiation therapy (PORT) before and after propensity score-matching (PSM). **a**, OS curves before PSM. The 5-year OS rate was 37.7% for the PORT group and 34.1% for the no PORT group. **b**, LCSS curves before PSM. The 5-year LCSS rate was 43.5% for the PORT group and 30.6% for the no PORT group. **c**, OS curves after PSM. The 5-year OS rate was 41.3% for the PORT group and 34.1% for the no PORT group. **d**, LCSS curves after PSM. The 5-year LCSS rate was 46.0% for the PORT group and 41.6% for the no PORT group

Before PSM, median and 5-year OS and LCSS values were significantly higher in patients who received PORT versus those who did not (median OS, 39 versus 35 months; 5-year OS, 37.7% versus 34.1%; *p* = 0.019 and median LCSS, 48 versus 41 months; 5-year LCSS, 43.5% versus 30.6%; *p* = 0.040) (Fig. 2a, b). Following PSM, OS and LCSS values were still significantly higher in patients who underwent PORT than in those who did not (median OS, 43 versus 34 months; 5-year OS, 41.3% versus 34.1%, *p* = 0.002 and median LCSS, 50 versus 41 months; 5-year LCSS, 46.0% versus 41.6%, *p* = 0.032) (Fig. 2c, d).

**Fig. 3:**
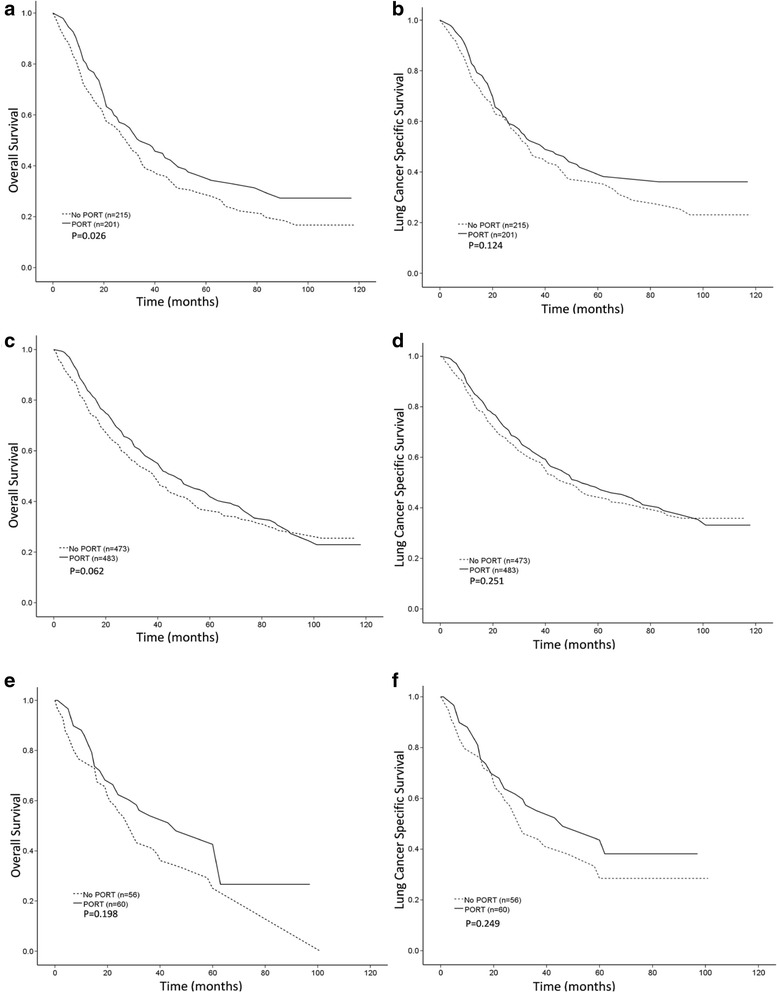
Overall survival (OS) and lung cancer-specific survival (LCSS) in patients with different age ranges according to the use of postoperative radiation therapy (PORT) after propensity score-matching (PSM). **a**, OS curves for patients aged <60 years. The 5-year OS rate was 35.4% for the PORT group and 28.9% for the no PORT group. **b**, LCSS curves forpatients aged <60 years. The 5-year LCSS rate was 39.5% for the PORT group and 36.2% for the no PORT group. **c**, OS curves for patients aged 60–79 years. The 5-year OS rate was 42.8% for the PORT group and 36.9% for the no PORT group. **d**, LCSS curves for patients aged 60–79 years. The 5-year LCSS rate was 48.2% for the PORT group and 45.0% for the no PORT group. **e**, OS curves for patients aged ≥80 years. The 5-year OS rate was 47.9% for the PORT group and 29.2% for the no PORT group. **f**, LCSS curves for patients aged ≥80 years. The 5-year LCSS rate was 49.0% for the PORT group and 33.3% for the no PORT group

In the age subgroup analysis after PSM, PORT offered an OS benefit only to patients aged < 60 years (5-year OS, 35.4% for PORT versus 28.9% for no PORT; *p* = 0.026). There was no significant difference in LCSS between the PORT and no PORT patients in the <60 years of age subgroup (Fig. 3a, b) or in either OS or LCSS between the PORT and no PORT patients in the 60–79 years of age and ≥80 years of age subgroups (Fig. 3c–f).

**Fig. 4:**
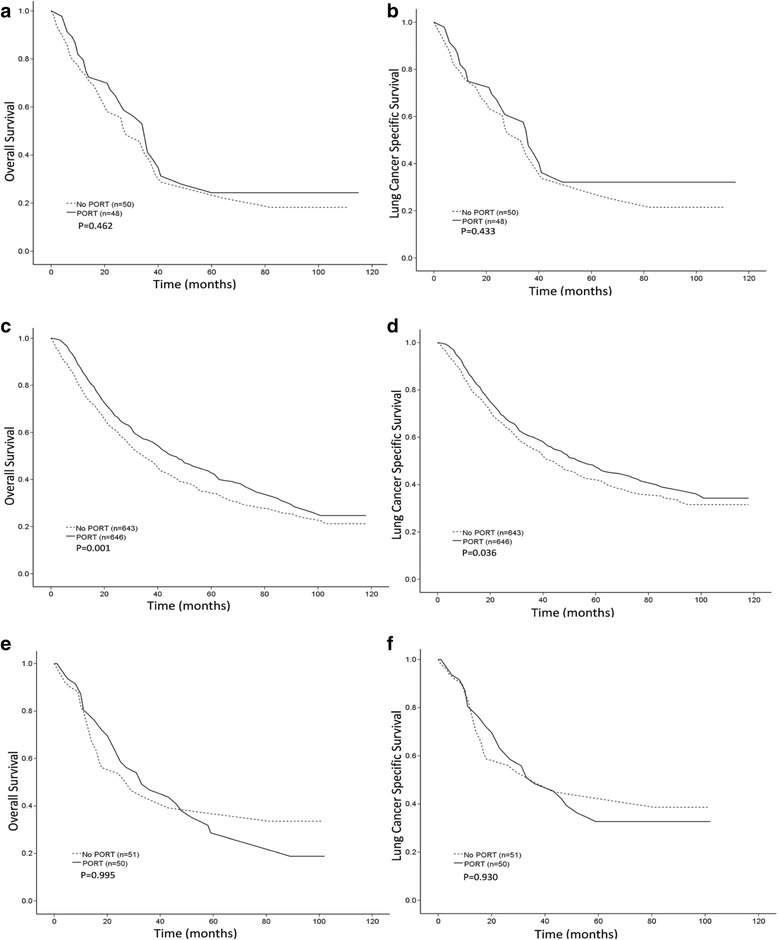
Overall survival (OS) and lung cancer-specific survival (LCSS) in patients who underwent different surgical procedures according to the use of postoperative radiation therapy (PORT) after propensity score-matching (PSM). **a**, OS curves for patients who underwent sublobectomy. The 5-year LCSS was 32.2% for the PORT group and 30.0% for the no PORT group. **b**, LCSS curves for patients who underwent sublobectomy. The 5-year LCSS rate was 27.8% for the PORT group and 25.6% for the no PORT group. **c**, OS curves for patients who underwent lobectomy. The 5-year OS rate was 43.5% for the PORT group and 34.5% for the no PORT group. **d**, LCSS curves for patients who underwent lobectomy. The 5-year LCSS rate was 48.3% for the PORT group and 42.3% for the no PORT group. **e**, OS curves for patients who underwent pneumonectomy. The 5-year OS rate was 28.7% for the PORT group and 39.1% for the no PORT group. **f**, LCSS curves for patients who underwent pneumonectomy. The 5-year LCSS rate was 32.7% for the PORT group and 45.1% for the no PORT group

In patients who received lobectomy, both OS and LCSS were better in the PORT versus the no PORT group (5-year OS, 43.5% versus 34.5%, *p* = 0.001 and 5-year LCSS, 48.3% versus 42.3%, *p* = 0.036) (Fig. 4c, ﻿d). OS and LCSS did not differ significantly between the patients in the PORT and no PORT groups who underwent sublobectomy or pneumonectomy (Fig. 4a, b, e, f).

Multivariate analysis revealed that the use of PORT was an independent prognostic factor for OS and LCSS both before and after PSM. Before PSM, the hazard ratio (HR) for PORT (compared with no PORT) was 0.846 (95% confidence interval [CI], 0.769–0.932; *p* = 0.001) for OS and 0.838 (95% CI, 0.755–0.931; *p* = 0.001) for LCSS. After PSM, the HR for PORT (compared with no PORT) was 0.793 (95% CI, 0.690–0.912; *p* = 0.001) and 0.837 (95% CI, 0.719–0.975; *p* = 0.022) for LCSS (Table 2). The other significant prognostic factors were age, tumor size, regional number of positive LNs, the ratio of positive to examined LNs, surgical procedure, and T stage.

**Table 2:** Multivariate analysis of predictors for OS and LCSS

Variable	Before PSM				After PSM			
	OS		LCSS		OS		LCSS	
	Hazard ratio (95% CI)	*p*	Hazard ratio (95% CI)	*p*	Hazard ratio (95% CI)	*p*	Hazard ratio (95% CI)	*p*
Age (versus <60 years)								
60–79 years	0.822 (0.742–0.911)	0.000	0.809 (0.723–0.904)	0.000	0.758 (0.648–0.887)	0.001	0.716 (0.604–0.850)	0.000
≥80 years	1.007 (0.844–1.201)	0.938	1.059 (0.878–1.277)	0.548	0.962 (0.730–1.267)	0.781	0.977 (0.727–1.313)	0.876
Gender (versus Male)	1.066 (0.974–1.168)	0.163	1.049 (0.951–1.158)	0.337	1.030 (0.895–1.186)	0.676	1.030 (0.884–1.202)	0.703
Race (versus White)	1.033 (0.923–1.157)	0.570	1.043 (0.922–1.179)	0.504	1.085 (0.912–1.291)	0.357	1.108 (0.917–1.339)	0.289
Marital status (versus Married)	1.001 (0.912–1.099)	0.984	0.966 (0.872–1.070)	0.511	1.160 (1.000–1.344)	0.049	1.173 (0.999–1.378)	0.052
Insurance (versus insured)	0.970 (0.883–1.065)	0.519	0.995 (0.899–1.101)	0.924	0.844 (0.730–0.977)	0.023	0.877 (0.748–1.027)	0.104
Laterality (versus left)	1.021 (0.929–1.121)	0.671	1.061 (0.958–1.175)	0.257	1.048 (0.906–1.212)	0.530	1.107 (0.943–1.298)	0.213
Location (versus upper lobe)								
Lower lobe	1.025 (0.812–1.294)	0.834	1.027 (0.800–1.317)	0.835	1.137 (0.807–1.601)	0.463	1.076 (0.737–1.570)	0.706
Middle lobe	1.059 (0.960–1.167)	0.251	1.024 (0.921–1.139)	0.660	0.996 (0.857–1.158)	0.961	0.958 (0.813–1.130)	0.612
Tumor size	1.005 (1.003–1.008)	0.000	1.006 (1.003–1.008)	0.000	1.007 (1.003–1.012)	0.000	1.009 (1.005–1.014)	0.000
Regional LN positive	1.034 (1.014–1.054)	0.001	1.040 (1.019–1.062)	0.000	1.053 (1.020–1.087)	0.002	1.069 (1.033–1.107)	0.000
Regional LN examined	0.995 (0.987–1.004)	0.261	0.993 (0.984–1.002)	0.136	0.994 (0.981–1.007)	0.370	0.988 (0.973–1.003)	0.121
% of LN positive	1.902 (1.490–2.429)	0.000	1.965 (1.507–2.562)	0.000	1.562 (1.041–2.342)	0.031	1.560 (0.997–2.439)	0.051
Histology (versus Adenocarcinoma)								
Squamous cell carcinoma	1.183 (1.058–1.323)	0.003	1.133 (1.002–1.282)	0.046	1.278 (1.077–1.516)	0.005	1.213 (1.004–1.467)	0.045
Adenosquamous and Large cell carcinoma	1.278 (1.069–1.527)	0.007	1.300 (1.073–1.575)	0.007	1.256 (0.964–1.637)	0.092	1.335 (1.007–1.771)	0.045
Differentiation grade (versus Well differentiated)								
Moderately differentiated	1.118 (0.901–1.387)	0.310	1.149 (0.906–1.458)	0.251	1.051 (0.703–1.570)	0.809	1.117 (0.717–1.741)	0.625
Poorly differentiated	1.245 (1.002–1.546)	0.048	1.284 (1.011–1.631)	0.041	1.168 (0.781–1.745)	0.449	1.238 (0.794–1.929)	0.346
Undifferentiated	1.371 (0.969–1.942)	0.075	1.377 (0.940–2.018)	0.101	1.434 (0.799–2.571)	0.227	1.526 (0.809–2.880)	0.192
Surgical procedure (versus Sublobectomy)								
Lobectomy	0.786 (0.664–0.930)	0.005	0.769 (0.641–0.922)	0.005	0.659 (0.500–0.868)	0.003	0.613 (0.456–0.825)	0.001
Pneumonectomy	0.778 (0.619–0.978)	0.032	0.757 (0.590–0.970)	0.028	0.515 (0.344–0.771)	0.001	0.487 (0.315–0.753)	0.001
T stage (sixth edition) (versus T1)								
T2	1.224 (1.090–1.375)	0.001	1.224 (1.078–1.390)	0.002	1.165 (0.971–1.397)	0.100	1.160 (0.950–1.417)	0.145
T3	1.816 (1.477–2.233)	0.000	1.960 (1.574–2.440)	0.000	1.747 (1.249–2.443)	0.001	1.812 (1.263–2.598)	0.001
PORT (versus No PORT)	0.846 (0.769–0.932)	0.001	0.838 (0.755–0.931)	0.001	0.793 (0.690–0.912)	0.001	0.837 (0.719–0.975)	0.022

*LN* lymph node, *PSM* propensity score-matching, *OS* overall survival, *LCSS* lung cancer specific survival, *PORT* postoperative radiotherapy, *CI* confidence interval

## Discussion

There is a high risk of both local and distant relapse after NSCLC resection. Adjuvant chemotherapy is typically administered after resection of stage II or III NSCLCs to reduce the possibility of recurrence and thus improve survival outcomes [15–17]. However, the rate of locoregional tumor recurrence is as high as 20%–40% even after adjuvant chemotherapy [2]. Therefore, studies have been performed to evaluate the effect of PORT on tumor recurrence and OS.

For completely resected N0 and N1 NSCLC, most studies have shown that PORT worsens survival [6, 7, 9, 18]. For N2 disease, the use of PORT is controversial owing to conflicting or inconclusive results in randomized studies performed before 1998 [3, 19–21]. Other studies showed that PORT improved local control and survival (the meta-analysis by Billiet et al. [13]), was more effective in patients with a high risk of local recurrence [22], and was mainly restricted to patients with multiple-station versus single-station N2 disease [23]. The randomized intergroup LungART trial, which is assessing the role of radiation after complete NSCLC resection, is ongoing with results not expected for several years [24].

Two retrospective studies using large population-based databases seemingly support the use of PORT for post-resection treatment of N2 NSCLC. Using data registered in the SEER database between 1988 and 2002, Lally et al. [6] found that PORT prolonged survival in patients with N2 NSCLC. A similar result was demonstrated in a population-based cohort study performed by Robinson et al. [5] using the NCDB. Although sample volumes were large and confounders were adjusted via multivariate analysis, bias in these two studies was not fully controlled. Therefore, conclusions should be drawn cautiously from the results.

Although propensity score methods may not fully eliminate confounding variables [25], they are often more practical and statistically more efficient in observational studies than are multivariate statistical methods [26]. An analysis of propensity score-matched patients can substitute in part for a randomized trial by directly comparing outcomes between individuals who received the treatment of interest and those who did not [27]. Some studies failed to demonstrate the superiority of propensity score methods compared with conventional multivariate regression analyses in terms of controlling confounders in specific situations [25, 28]. Nonetheless, the use of PSM in this study provides new information about the effects of PORT in patients with N2 NSCLC.

The results of our study show a modest improvement in the 5-year OS (3.6%) and LCSS (2.9%) rates in the PORT versus no PORT group before PSM. These improvements are less than those reported by Lally et al. [6], whose data were also derived from the SEER database, although from a different time period. The reasons for the discrepancies between the two studies are unknown, but may include the evolution in surgical and radiation techniques between 1988–2002 and 2004–2013 and disparity in the inclusion criteria. PORT-related improvements in the 5-year OS and LCSS rates were slightly higher after PSM than before PSM. This finding indicates that the bias of the baseline variables skews the results toward null.

In the age subgroup analysis, there was modest OS superiority of PORT over no PORT in younger patients (age < 60 years), which disappeared gradually as the age of the patients increased. This result may reflect the good performance status of the younger patients and their capability to better survive the cardiac and pulmonary complications of radiotherapy. PORT did not significantly improve LCSS in any of the age subgroups, which indicates that more patients died of non-cancer causes in the no PORT group than the PORT group. It is reasonable to presume that there were more comorbidities in the no PORT group, which contributed to the observed results.

Regarding surgical procedures, PORT improved OS and LCSS in patients who underwent lobectomy but not sublobectomy or pneumonectomy. This finding indicates that patients who receive sublobectomy or pneumonectomy should avoid radiation. The toxicity of PORT following pneumonectomy may offset its benefits. We speculate that the patients who received sublobectomy may have had poor cardiopulmonary function or severe comorbidities that contraindicated lobectomy and pneumonectomy and decreased tolerance for PORT. These possibilities may account for the lack of a positive effect of PORT on survival in cases involving sublobectomy.

Our multivariate analysis showed that use of PORT was an independent prognostic factor for OS and LCSS, both before and after PSM. Some other well-established predictors for poorer survival were also confirmed in this study, including increased tumor size, a large number of regional positive LNs, the percentage of positive LNs, squamous cell carcinoma, and sublobectomy [6, 29–32].

This study has the typical limitations of a retrospective study. Selection bias cannot be fully eliminated even after PSM because this method is based on the available variables, and unadjusted confounding factors may still exist [26]. Moreover, the SEER database is itself a limitation because observational data may engender inaccurate results [33]. Additional limitations of the SEER database are as follows. First, there was no information regarding the surgical margin status. Compared with a negative surgical margin, a positive surgical margin increases the risk of locoregional recurrence, thus decreasing OS rates, and tends to lead to the use of PORT. This confounder would bias the result toward a null result. Second, the performance status and comorbidities of the patients were unknown. Usually, patients with a good performance status are more likely to receive PORT, leading to a result favoring the use of PORT. Third, there was no information regarding the use of systemic therapies such as chemotherapy and targeted treatment. Chemotherapy and targeted therapy are strong prognostic factors for NSCLC and can influence the prescription and results of PORT [34]. Four, there was no information about PORT parameters (e.g., dose, segmentation, and use of a linear accelerator or cobalt) that would certainly affect the treatment results [13]. Lastly, detailed information regarding surgical complications was lacking. Severe surgical complications will limit the use of the PORT and also affect survival.

## Conclusions

Our analysis of the SEER database using PSM to reduce selection bias demonstrates that PORT has a significant survival benefit for patients with N2 NSCLC. However, the advantage is only modest. Unlike previous studies, in which PORT positively affected patients with N2 disease regardless of age or treatment [5, 6], our study suggests that PORT mainly benefits younger patients (age < 60 years) and those who underwent lobectomy as opposed to pneumonectomy or sublobectomy. Owing to the retrospective nature of this study, prospective randomized evidence is needed to further clarify the efficacy of PORT for treatment of N2 NSCLC.

## Abbreviations

CI: Confidence interval
HR: Hazard ratio
LCSS: Lung cancer-specific survival
LN: Lymph node
NSCLC: Non-small cell lung cancer
OS: Overall survival
PORT: Postoperative radiotherapy
PSM: Propensity score-matching
SEER: Surveillance, epidemiology, and end results
